# Inhibition of histone methyltransferase G9a attenuates liver cancer initiation by sensitizing DNA-damaged hepatocytes to p53-induced apoptosis

**DOI:** 10.1038/s41419-020-03381-1

**Published:** 2021-01-19

**Authors:** Takuma Nakatsuka, Keisuke Tateishi, Hiroyuki Kato, Hiroaki Fujiwara, Keisuke Yamamoto, Yotaro Kudo, Hayato Nakagawa, Yasuo Tanaka, Hideaki Ijichi, Tsuneo Ikenoue, Takeaki Ishizawa, Kiyoshi Hasegawa, Makoto Tachibana, Yoichi Shinkai, Kazuhiko Koike

**Affiliations:** 1 Department of Gastroenterology, Graduate School of Medicine, The University of Tokyo, 7-3-1 Hongo, Bunkyo-ku, Tokyo 113-8655 Japan; 2 Division of Gastroenterology, The Institute for Adult Diseases, Asahi Life Foundation, 2-2-6 Bakurocho, Chuo-ku, Tokyo 103-0002 Japan; 3 Division of Clinical Genome Research, Advanced Clinical Research Center, Institute of Medical Science, The University of Tokyo, 4-6-1 Shirokanedai, Minato-ku, Tokyo 108-8639 Japan; 4 Hepato-Biliary-Pancreatic Surgery Division, Department of Surgery, Graduate School of Medicine, The University of Tokyo, 7-3-1 Hongo, Bunkyo-ku, Tokyo 113-8655 Japan; 5 Laboratory of Epigenome Dynamics, Graduate School of Frontier Biosciences, Osaka University, 1-3 Yamadaoka, Suita, 565-0871 Japan; 6 Cellular Memory Laboratory, RIKEN Cluster for Pioneering Research, 2-1 Hirosawa, Wako, Saitama 351-0198 Japan

**Keywords:** Liver cancer, Apoptosis, Gene silencing, Liver cancer, Experimental models of disease

## Abstract

While the significance of acquired genetic abnormalities in the initiation of hepatocellular carcinoma (HCC) has been established, the role of epigenetic modification remains unknown. Here we identified the pivotal role of histone methyltransferase G9a in the DNA damage-triggered initiation of HCC. Using liver-specific *G9a*-deficient (*G9a*^ΔHep^) mice, we revealed that loss of G9a significantly attenuated liver tumor initiation caused by diethylnitrosamine (DEN). In addition, pharmacological inhibition of G9a attenuated the DEN-induced initiation of HCC. After treatment with DEN, while the induction of γH2AX and p53 were comparable in the *G9a*^ΔHep^ and wild-type livers, more apoptotic hepatocytes were detected in the *G9a*^ΔHep^ liver. Transcriptome analysis identified Bcl-G, a pro-apoptotic Bcl-2 family member, to be markedly upregulated in the *G9a*^ΔHep^ liver. In human cultured hepatoma cells, a G9a inhibitor, UNC0638, upregulated BCL-G expression and enhanced the apoptotic response after treatment with hydrogen peroxide or irradiation, suggesting an essential role of the G9a-Bcl-G axis in DNA damage response in hepatocytes. The proposed mechanism was that DNA damage stimuli recruited G9a to the p53-responsive element of the *Bcl-G* gene, resulting in the impaired enrichment of p53 to the region and the attenuation of Bcl-G expression. G9a deletion allowed the recruitment of p53 and upregulated Bcl-G expression. These results demonstrate that G9a allows DNA-damaged hepatocytes to escape p53-induced apoptosis by silencing Bcl-G, which may contribute to the tumor initiation. Therefore, G9a inhibition can be a novel preventive strategy for HCC.

## Introduction

Hepatocellular carcinoma (HCC) accounts for the majority of primary liver cancers and is the fourth most common cause of cancer-related deaths worldwide^1^. HCC occurs due to a variety of risk factors, including viral hepatitis, alcoholic and nonalcoholic fatty liver disease, carcinogen exposure, and metabolic liver diseases. Despite recent advances in the pathophysiology and treatment of HCC, the overall prognosis remains poor. A better understanding of the mechanisms of hepatocarcinogenesis will provide novel and efficacious targets for treatment of HCC.

HCC develops through a complex multistep process in which various genomic abnormalities are acquired. In addition to genetic alterations, accumulating evidence has highlighted the key role of epigenetic dysregulation in liver cancer pathogenesis^2^. Consistently, recent genome analysis has revealed a high number of genetic disturbances in the genes related to epigenetics in HCC^3,4^. Global DNA hypomethylation and promoter hypermethylation of specific tumor suppressor genes are well-characterized epigenetic changes in human carcinogenesis^5^. In addition to DNA methylation, aberrant expression of histone modifiers has also been implicated in the tumor development. Histone methylation critically determines the chromosomal structure as well as accessibility to transcription factors.^6^ Disturbances in histone methylation associate with HCC development^7–9^. Therefore, modulating the enzymatic activities of histone modifiers might be a potential therapeutic strategy to prevent HCC.

G9a (also known as euchromatic histone-lysine N-methyltransferase 2, EHMT2) is a SET domain-containing protein that catalyzes specifically the di-methylation of histone 3 lysine 9 (H3K9me2), which is a prominent epigenetic marker of transcriptional repression^10^. G9a plays important roles in diverse cellular processes, such as proliferation, differentiation, senescence, and autophagy^11^. The dysregulation of G9a and aberrant levels of H3K9me2 are involved in different types of human cancers^12–15^. As for HCC, G9a promotes tumor progression by silencing tumor suppressor genes or enhancing epithelial-mesenchymal transition^16,17^, and its inhibition reduces tumor aggressiveness^18,19^. In contrast, how G9a contributes to the development or initiation of HCC has not yet been investigated in vivo.

In chronic hepatic injury caused by viral infection or excessive fat accumulation, DNA damage from produced reactive oxygen species is a critical pathogenic factor for liver carcinogenesis^20^. Given the role of G9a for DNA damage repair and cell survival^21^, we hypothesized that G9a might be involved in DNA damage-induced liver cancer initiation. In this study, we demonstrate that liver-specific *G9a*-deficient (*G9a*^ΔHep^) mice suppress HCC development triggered by diethylnitrosamine (DEN) and the pivotal role of G9a in DNA damaged hepatocytes.

## Results

### G9a is frequently upregulated in human HCC

**Fig. 1:**
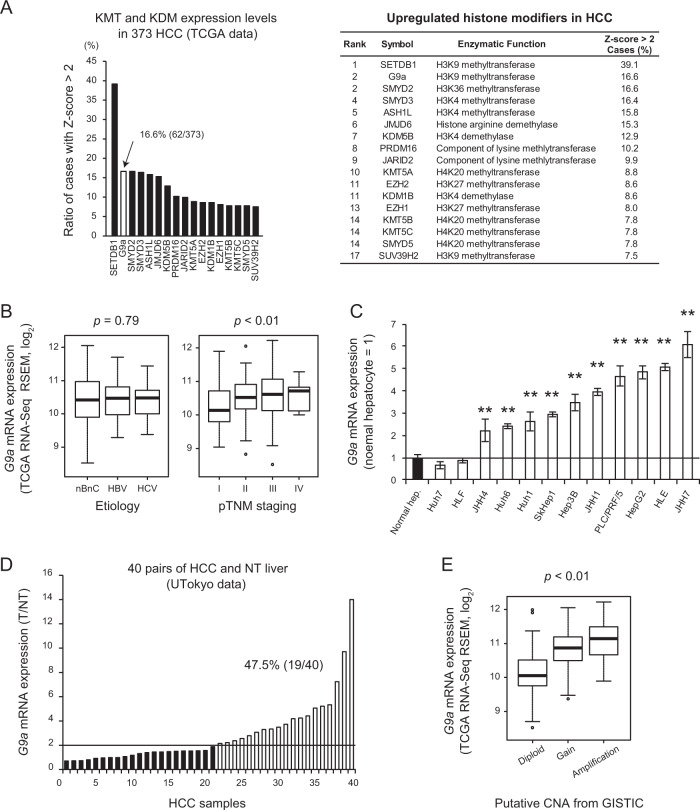
*G9a* is highly expressed in human HCC. **A** Relative expression levels of histone-lysine methyltransferase (KMT) and demethylase (KDM) in hepatocellular carcinoma using The Cancer Genome Atlas (TCGA) data set. **B** *G9a* expression is comparable among etiologies (nBnC, *n* = 180; HBV, *n* = 97; HCV, *n* = 49; *p* = 0.79, one-way ANOVA) and increases according to the pTNM stage in the TCGA cohort (Stage I, *n* = 171; II, *n* = 85; III, *n* = 85; IV, *n* = 5; *p* < 0.01, one-way ANOVA). The top and bottom boxes represent the first and third quartiles, respectively. The thick line inside each box represents the median. Whiskers represent 1.5 interquartile ranges from the hinges. Open circles represent outliers. **C** *G9a* is upregulated in many HCC cell lines. Data are mean ± SEM from three independent experiments. **p < 0.01, Student’s *t* test. **D** *G9a* expression levels in 40 pairs of HCC (T) and surrounding non-tumorous (NT) liver obtained in our institution. *G9a* expression level is more than double in the tumor in 19 of 40 cases (47.5%). **E** *G9a* gene copy number is positively correlated with mRNA expression levels in the TCGA data set (*p* < 0.01, one-way ANOVA).

First, we analyzed the expression levels of histone modifiers from 373 HCC cases identified in The Cancer Genome Atlas (TCGA) dataset. From the 82 identified histone modifiers, many histone methyltransferases were upregulated; consistent with previous papers, *SETDB1* was the most significantly upregulated^9,22^ (Figs. 1A, S1). *G9a* was also identified as one of the most upregulated genes, with high expression levels (Z-score > 2) in 16.6% (62/373) of the cases (Fig. 1A). Expression levels of *G9a* were not significantly different among etiologies but rather tended to increase according to the pathological malignancy grade of HCC (Fig. 1B). *G9a* expression was also higher in many HCC cell lines than in normal hepatocytes (Fig. 1C). In addition, in an original cohort consisting of 40 pairs of HCC and adjacent NT livers in our institution, we successfully validated the upregulation of G9a in HCC compared with NT livers in almost half of the cases (Fig. 1D). In the TCGA cohort, copy number gain of *G9a* gene was detected in 4.29% (16/373) of the cases, and it was positively correlated with higher *G9a* mRNA expression levels (Fig. 1E). These data confirm the hypothesis that accumulated genomic alterations in advanced HCC are linked to *G9a* upregulation as well as *SETDB1*^16,22^, and highlight the pathological significance of G9a in HCC. Of note, given the enzymatic roles of G9a and SETDB1 related to H3K9 methylation, the above findings suggest the molecular significance of H3K9 methylation in HCC pathogenesis. In contrast, however, the involvement of G9a and its H3K9 methylation activity in hepatocarcinogenesis in vivo remains unclear. Thus, we next examined the significance of G9a in liver tumor development using liver-specific *G9a*-deficient (*G9a*^ΔHep^) mice.

### Loss of G9a attenuates liver tumorigenesis in a carcinogen-induced HCC model

**Fig. 2:**
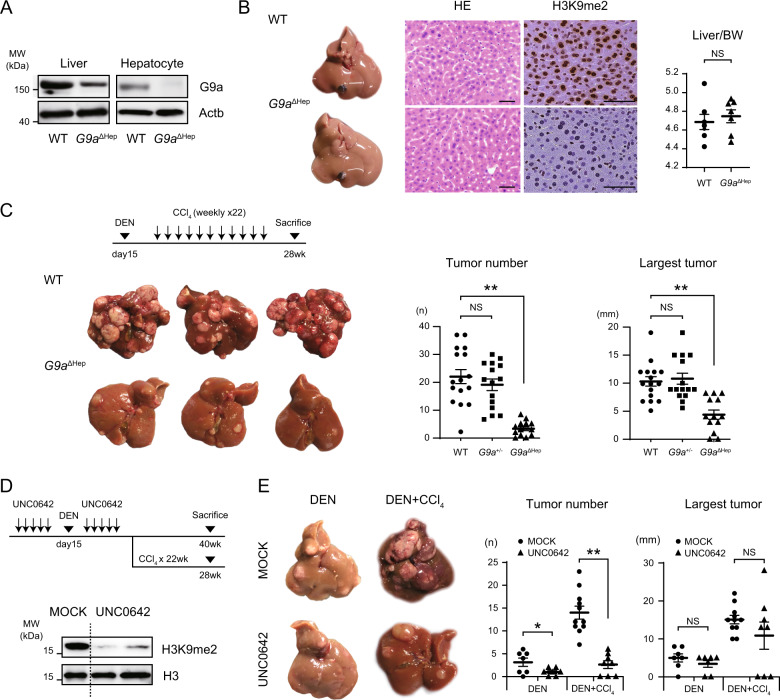
*G9a* inhibition attenuates DEN-induced hepatocarcinogenesis. **A** G9a deletion is confirmed using immunoblotting of liver lysates and isolated primary hepatocytes. **B** Representative macro- and micro-images of the liver and liver/body weight (BW) ratio of 30-week-old mice. There is no obvious difference between wild-type (WT) and *G9a*^ΔHep^ livers, but H3K9me2 levels are markedly attenuated in the *G9a*^ΔHep^ hepatocytes. Scale bars, 50 µm; each, *n* = 7; NS not significant, Student’s *t* test. **C** WT and *G9a*^ΔHep^ mice are subjected to diethylnitrosamine (DEN) (25 mg/kg i.p.) at day 15 postpartum, followed by 22 injections of carbon tetrachloride (CCl_4_) (0.5. mL/kg i.p.) per week and euthanized 6.5 months after DEN administration. Liver tumor development is significantly attenuated in *G9a*^ΔHep^ mice (WT, *n* = 16; *G9a*^+/−^, *n* = 15; *G9a*^ΔHep^, *n* = 13; **p < 0.01, NS not significant, Student’s *t* test). **D** Immunoblots using liver lysates after five sequential days of UNC0642 injection reveal that G9a inhibitor successfully reduces H3K9me2 levels in the liver. **E** UNC0642 treatment significantly attenuates DEN-induced liver tumor development (DEN/MOCK, *n* = 7; DEN/UNC0642, *n* = 7; DEN + CCl_4_/MOCK, *n* = 11; DEN + CCl_4_/UNC0642, *n* = 8; *p < 0.05, **p < 0.01, NS not significant, Student’s *t* test).

*G9a*^ΔHep^ mice were established by crossing *G9a*^flox/flox^ mice with albumin-Cre transgenic mice. To confirm efficient deletion of G9a in hepatocytes, the expression levels of G9a protein were measured in whole liver and isolated primary hepatocytes. Deletion of G9a was confirmed in *G9a*^ΔHep^ mouse hepatocyte, while residual G9a expression was detected in the whole liver lysate of *G9a*^ΔHep^ mice, probably from nonhepatocyte fractions (Fig. 2A). Immunohistochemistry revealed that H3K9me2 levels in *G9a*^ΔHep^ mouse hepatocytes nuclei decreased significantly, suggesting the critical role of G9a in the H3K9me2 modification (Fig. 2B). *G9a*^ΔHep^ mice lineage was consistent with Mendel’s laws of inheritance, and there were no significant issues with their growth. As for the liver, there were no differences in liver weight or histology between *G9a*^ΔHep^ and wild-type (WT) mice (Fig. 2B).

To address the role of G9a in liver carcinogenesis, we injected a combination of DEN and hepatotoxin carbontetrachloride (CCl_4_) solution into WT and *G9a*^ΔHep^ mice. The chemical carcinogenic model mimics chronic inflammation and fibrosis of the human liver, and the developed tumor has the characteristics of human HCC^23^. In the inflammation-related liver-tumorigenic setting, *G9a*^ΔHep^ mice displayed a profound reduction in tumor number and size (Fig. 2C). *G9a*^ΔHep^ mice did not show significant changes in the pathology, proliferation, and apoptosis of the barely formed tumors (Fig. S2A), which leads us to suggest that *G9a* is critical in the initial stage of liver tumorigenesis. Supportive of this notion, decreased liver tumor initiation in *G9a*^ΔHep^ mice was also observed under a purely genotoxic carcinogenic protocol without inflammation, namely, simple DEN administration without CCl_4_ injection (Fig. S2B).

To confirm the tumor-initiating role for G9a, we investigated the effects of pharmacological inhibition of G9a in the above in vivo settings. Treatment with the G9a inhibitor UNC0642 effectively reduced H3K9me2 levels in liver cells (Fig. 2D). Notably, treatment with UNC0642 resulted in significant attenuation of DEN-induced liver tumor development, regardless of subsequent CCl_4_ administration (Fig. 2E). In contrast, the size of largest tumors was not efficiently affected by the administration of UNC0642 for 10 days, suggesting that the inhibition of G9a may predominantly affect the tumor initiation. These findings support our hypothesis that the enzymatic function of G9a is involved in the liver tumor initiation following DEN administration.

### G9a deletion induced apoptosis in DNA-damaged hepatocytes

**Fig. 3:**
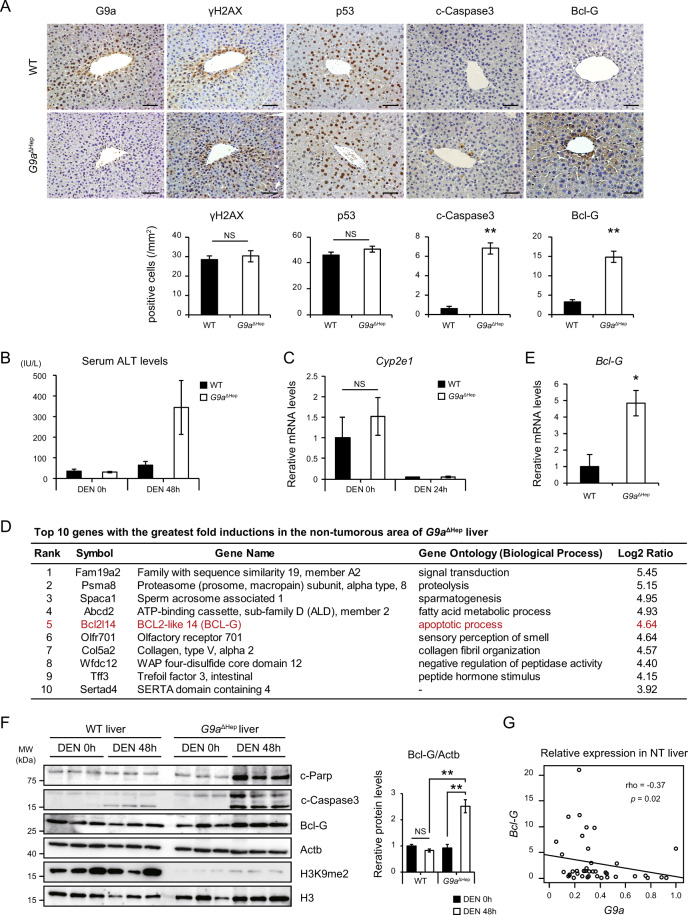
*G9a* protects hepatocytes against apoptosis after a high dose of DEN. **A** Immunohistochemical analysis of the mice liver 48 h after administering a high dose of diethylnitrosamine (DEN) (100 mg/kg i.p.). G9a is upregulated after DEN administration, and cleaved-Caspase 3 induction occurs only in the *G9a*^ΔHep^ liver, whereas γH2AX and p53 levels are comparable between wild-type (WT) and *G9a*^ΔHep^ livers. Bcl-G expression is elevated in the pericentral hepatocytes of *G9a*^ΔHep^ mice. Scale bars, 50 µm. The number of positive hepatocytes is shown in the graph below (**p < 0.01, NS not significant, Student’s *t* test). **B** Serum ALT levels are significantly elevated in *G9a*^ΔHep^ mice 48 h after administering a high dose of DEN. Data are represented as mean ± SEM (WT/DEN0h, *n* = 4; *G9a*^ΔHep^/DEN0h, n = 3; WT/DEN48h, *n* = 5; *G9a*^ΔHep^/DEN48h, *n* = 3). **C** *Cyp2e1* expression, as assessed by qRT-PCR, is comparable between WT and *G9a*^ΔHep^ livers (each, *n* = 3; NS not significant, Student’s *t* test). **D** Top 10 upregulated genes in the non-tumorous area of *G9a*^ΔHep^ liver are listed. **E** qRT-PCR analysis validates *Bcl-G* upregulation in the *G9a*^ΔHep^ liver (*p < 0.05, Student’s *t* test). **F** Immunoblots reveal increased Bcl-G, cleaved-PARP, and cleaved-Caspase3 protein levels in the *G9a*^ΔHep^ liver 48 h after high-dose DEN administration. Right graph shows relative protein level of Bcl-G (each, *n* = 3; **p < 0.01, NS not significant, Student’s *t* test). **G** Relative expression levels of *G9a* and *BCL-G* in human non-tumorous (NT) liver obtained in our institution. *BCL-G* expression is negatively correlated with *G9a* expression. (rho = −0.37, *p* = 0.02, Spearman’s rank correlation coefficient).

DEN, a DNA-damaging carcinogen, is metabolized and activated to form DNA adducts mainly in Cyp2E1-expressing pericentral hepatocytes^24^. DNA damage caused by DEN is a significant factor for subsequent HCC development^25^. To assess the implication of G9a in DNA damage-triggered tumor initiation, both WT and *G9a*^ΔHep^ mice were injected with a higher dose of DEN and were euthanized 48 h post-injection. Expression of the G9a protein was predominantly observed around the central vein in WT hepatocytes after DEN administration (Fig. 3A), suggesting that G9a is upregulated in DNA-damaged hepatocytes. Although the levels of DNA damage, detected by γH2AX staining and p53 induction, were identical in WT and *G9a*^ΔHep^ mice livers, higher numbers of cleaved-Caspase 3-positive hepatocytes were observed around the central vein in *G9a*^ΔHep^ mice than in WT mice (Fig. 3A). The serum levels of alanine aminotransferase (ALT) after DEN injection were markedly higher in *G9a*^ΔHep^ mice than in WT mice (Fig. 3B). To exclude the possibility that the chemical activity of DEN was enhanced by G9a deletion, we examined the expression of *Cyp2e1*. As reported previously, *Cyp2e1* was downregulated after DEN treatment in WT mice liver^26^. As we did not observe any significant difference in *Cyp2e1* levels between WT and *G9a*^ΔHep^ liver, we suggest that G9a deletion affects the cellular response against DEN-induced DNA damage, but not the chemical activity of DEN, in pericentral hepatocytes (Fig. 3C).

Given the epigenetic function of G9a as a transcriptional repressor, we hypothesized that G9a might regulate the expression of genes which determine cellular responses under DNA damage. To identify such genes regulated by G9a, comprehensive gene expression profiles of NT areas were analyzed in WT and *G9a*^ΔHep^ livers. Based on the function of G9a as a transcriptional repressor, we focused on the genes upregulated in the *G9a*^ΔHep^ liver. The top ten genes with the greatest fold inductions in *G9a*^ΔHep^ liver are shown in Fig. 3D. Among them, Bcl2-like 14 (also known as Bcl-G), a pro-apoptotic Bcl-2 family member, was significantly upregulated in the *G9a*^ΔHep^ liver. Quantitative real-time PCR confirmed that *Bcl-G* mRNA expression levels increased significantly in the NT areas of *G9a*^ΔHep^ liver (Fig. 3E). Immunoblotting revealed that Bcl-G protein, together with cleaved-PARP and cleaved-Caspase 3, accumulate after DEN administration in *G9a*^ΔHep^ liver but not in WT liver (Fig. 3F). In addition, immunohistochemistry showed that Bcl-G expression is markedly elevated in pericentral hepatocytes of *G9a*^ΔHep^ mice (Fig. 3A). These findings suggest that G9a-dependent transcriptional regulation of *Bcl-G* gene suppresses apoptosis under DNA damage in murine liver. Furthermore, to study the relationships in the expression of *G9a* and *Bcl-G* gene, we measured the levels of *G9a* and *BCL-G* mRNA in human NT liver samples. Supporting the findings in murine liver, the expression levels of *BCL-G* were negatively correlated to that of *G9a* in human NT liver (Fig. 3G).

### G9a inhibits DNA damage-induced apoptosis by regulating BCL-G expression

**Fig. 4:**
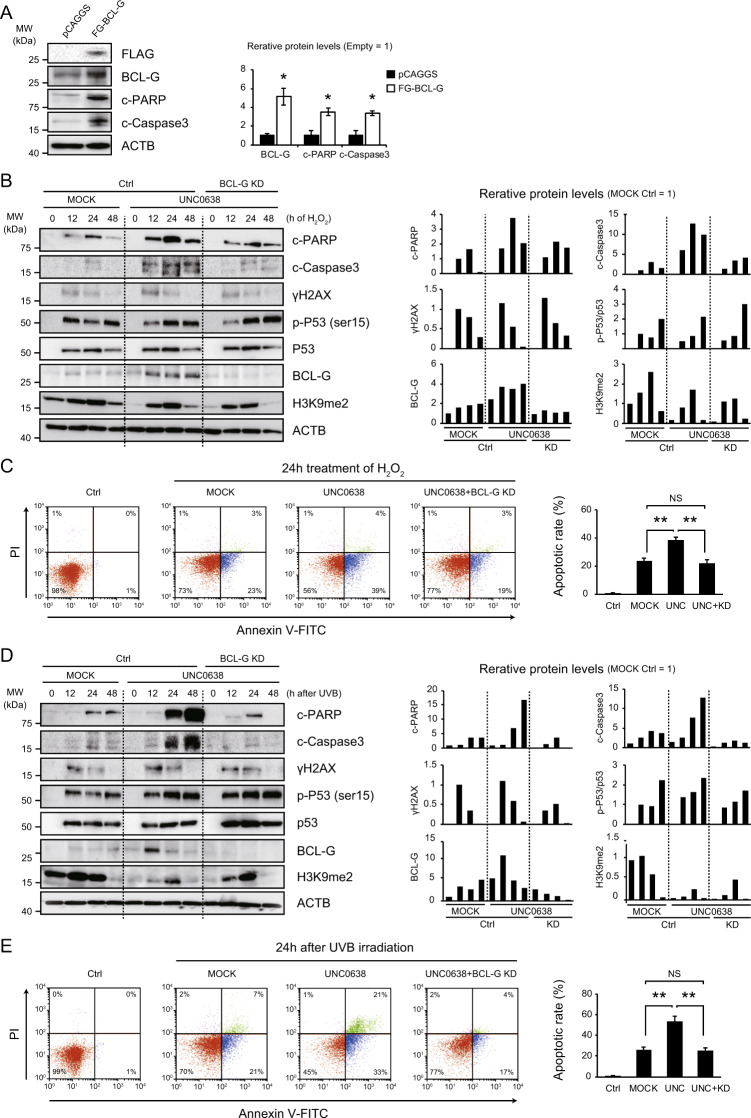
BCL-G is involved in apoptosis during the DNA damage response in hepatocytes. **A** BCL-G overexpression induces apoptotic changes in normal human hepatocytes. Representative immunoblot images and relative expression levels of the proteins are shown (each, *n* = 3; *p < 0.05, Student’s *t* test). **B**, **D** The G9a inhibitor UNC0638 (5 µM) promotes apoptosis in hepatocytes after treatment with hydrogen peroxide (H_2_O_2_, 2 mM) or UVB irradiation (300 J/m^2^) with an increase in BCL-G expression. Bcl-G knockdown (KD) suppresses the excessive induction of apoptosis caused by a G9a inhibitor. Right graphs show quantification of relative protein levels. **C**, **E** Representative dot-plot diagrams showing the percentage of the hepatocyte lines stained with Annexin V-FITC and propidium iodide (PI) after 24 h of treatment with H_2_O_2_ or UVB irradiation. The results of three independent experiments are shown in the right graph (**p < 0.01, NS not significant, Student’s *t* test).

We hypothesized that the regulation of *Bcl-G* expression by G9a might be a gatekeeper of apoptosis in DNA-damaged hepatocytes. To prove this hypothesis, we first examined the induction of apoptosis after BCL-G overexpression in a normal human hepatocyte cell line. BCL-G overexpression induced the cleavage of PARP and Caspase3, suggesting the pro-apoptotic role of BCL-G in human hepatocytes (Fig. 4A).

Next, we examined if the G9a-BCL-G axis is involved in apoptosis caused by DNA damage in the hepatocytes. For this purpose, the hepatocyte lines were treated with hydrogen peroxide (H_2_O_2_, 2 mM) or UVB irradiation (300 J/m^2^), which are well-known DNA damage inducers. As expected, both H_2_O_2_ administration and UVB irradiation increased total p53 and phosphorylated p53 levels, the active form often induced by DNA damage^27^, followed by the upregulation of cleaved-PARP and cleaved-Caspase3 (Fig. 4B, D). Treatment with 5 µM of a G9a inhibitor UNC0638 significantly attenuated the global levels of H3K9me2 in the hepatocyte lines (Fig. S3A), without affecting cell cycle status (Fig. S3B). In order to examine the role of G9a in DNA-damaged hepatocyte, the hepatocyte lines were treated with 5 µM of UNC0638 for 24 h before treatment with DNA damage inducers. In contrast to the slight increase of BCL-G after the introduction of DNA damage inducers in the control hepatocytes, the G9a inhibitor UNC0638 strikingly enhanced the expression of BCL-G (Fig. 4B, D). UNC0638 also induced PARP or Caspase3 cleavage while it did not always affect p53 activation, and it was suppressed in BCL-G knockdown cells (Fig. 4B, D). The enhancement of apoptotic changes by G9a inhibitor and their attenuation by BCL-G knockdown were also confirmed by flow cytometric analysis (Fig. 4C, E). These findings indicate that BCL-G, induced by G9a inhibition, is involved in the induction of apoptosis in DNA-damaged hepatocytes. The global levels of H3K9me2 increased after both treatments, and decreased, at least in part, by UNC0638, indicating that G9a contributes to DNA damage-induced H3K9me2 modification in hepatocytes. The residual H3K9me2 marks imply the involvement of other histone modifiers for H3K9me2.

### G9a regulates Bcl-G expression by mediating p53 recruitment to its response element

**Fig. 5:**
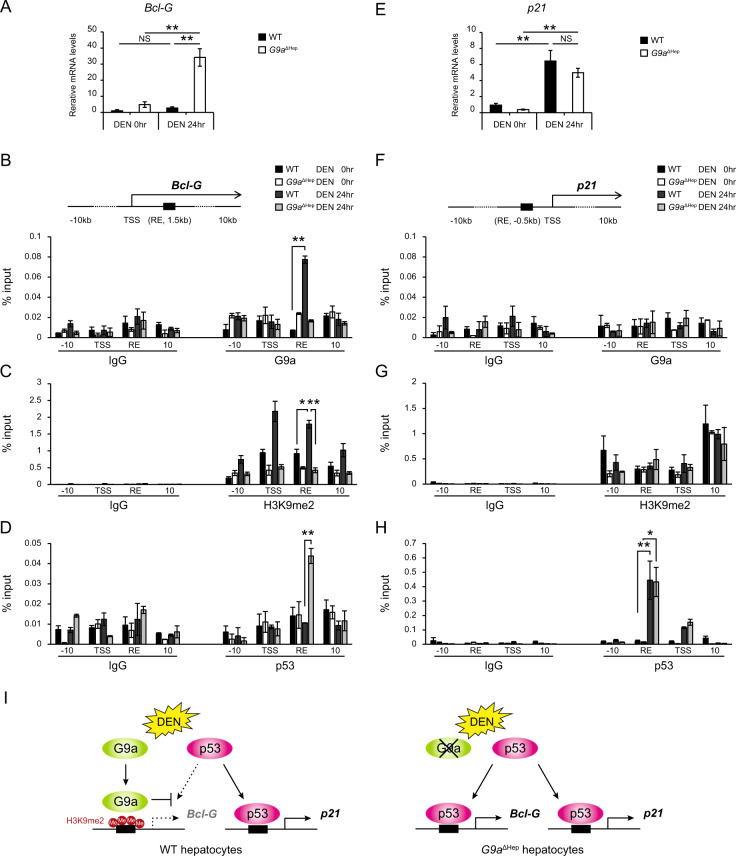
G9a inhibits p53 recruitment to its response element on Bcl-G in an H3K9 methylase-dependent manner. **A**, **E** Relative expression levels of Bcl-G and p21 are determined by qRT-PCR in WT and *G9a*^ΔHep^ mice livers before and after a high dose of diethylnitrosamine (DEN) administration (each, *n* = 3; **p < 0.01, NS not significant, Student’s *t* test). **B**–**D**, **F**–**H** Chromatin immunoprecipitation analyzes G9a, p53, and H3K9me2 binding at the indicated loci of *Bcl-G* and *p21*. The recruitment of G9a and increased H3K9me2 levels are shown in the *Bcl-G* gene (**B**, **C**), but not in the *P21* gene (**F**, **G**) after a high dose of DEN. *G9a* ablation, combined with DEN treatment, allows p53 recruitment onto the *Bcl-G* locus via a reduction in H3K9me2 levels (**D**). In contrast, p53 is recruited onto the *p21* locus after DEN treatment, independent of *G9a* status (**H**). Transcriptional start site (TSS), p53 response element (RE), and amplicons (10 kb upstream or downstream regions of TSS) are shown in the above diagrams. Results are expressed as the mean ± SEM of three independent experiments. *p < 0.05, **p < 0.01, Student’s *t* test. **I** Diagram showing the regulation of p53 target gene expression by G9a. G9a inhibits p53 binding to RE of *Bcl-G* gene in an H3K9me2-dependent manner and suppresses Bcl-G expression. G9a deletion enables p53 recruitment to *Bcl-G* gene RE and upregulates Bcl-G expression.

*Bcl-G* is a p53 direct target gene.^28^ Notably, UNC0638 altered BCL-G expression without affecting p53 activity in DNA-damaged hepatocytes (Fig. 4B, C). In addition, although p53 activation was comparable between WT and *G9a*^ΔHep^ mice livers after administering a higher dose of DEN (Fig. 3A), Bcl-G protein and gene expression levels were significantly higher in the liver of *G9a*^ΔHep^ mice (Figs. 3G and 5A). These findings suggest that G9a regulates Bcl-G expression without affecting p53 status in DNA-damaged hepatocytes. Histone modifiers regulate gene expression by mediating the recruitment of transcriptional machinery to their target genes.^29–31^ Therefore, to determine the mechanism through which G9a regulates the expression of Bcl-G, we first examined the binding of G9a to the p53 response element (RE) on the *Bcl-G* gene using the chromatin immunoprecipitation (ChIP) assay in mice livers. G9a was significantly enriched in the p53 RE of the *Bcl-G* gene in the WT liver after administration of a high dose of DEN, while it did not bind to the p53 RE in non-treated WT liver cells (Fig. 5B). H3K9me2 levels at the p53 RE were elevated in the WT liver after DEN administration, but it remained unchanged in the *G9a*^ΔHep^ liver (Fig. 5C). Importantly, while p53 binding to the RE of the *Bcl-G* gene was not detected in the WT liver, *G9a* deletion increased significantly the recruitment of p53 to the RE of the *Bcl-G* gene after DEN administration (Fig. 5D). Thus, G9a might regulate Bcl-G expression by interfering with the p53 recruitment in an H3K9 methylase-dependent manner.

Next, we examined whether the regulation of p53 recruitment by G9a is a common phenomenon for p53 target genes. We focused on p21, one of the major target genes of p53, because it is well known that DEN induces p53-dependent p21 upregulation.^32^ High doses of DEN significantly upregulated the expression of p21, and importantly, the upregulation was independent of G9a status (Fig. 5E). The ChIP assay revealed that G9a did not bound to the p53 RE of the *p21* gene after DEN administration (Fig. 5F). Furthermore, the levels of H3k9me2 were not elevated following DEN administration (Fig. 5G), and p53 was enriched in the p53 RE of the *p21* gene in both WT and *G9a*^ΔHep^ liver (Fig. 5H). These findings indicate that G9a is not involved in the regulation of p21 expression. Thus, G9a individually regulates p53 target genes expression through selective control of p53 recruitment (Fig. 5I).

## Discussion

**Fig. 6:**
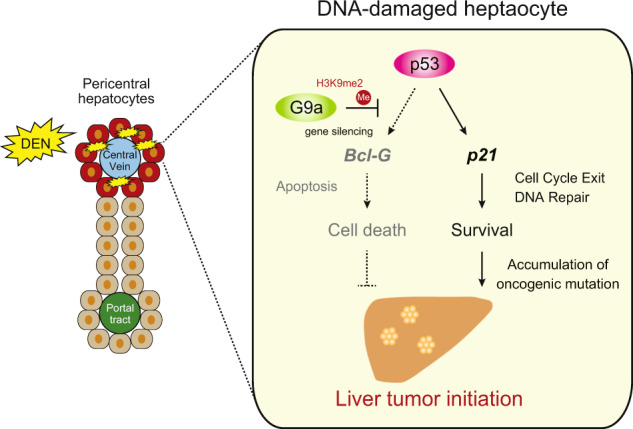
Graphical scheme. Schematic representation of the *G9a*-p53-Bcl-G circuit that controls liver tumor initiation from DNA-damaged hepatocytes. DEN-exposed pericentral hepatocytes undergo DNA damage and mutagenesis. G9a allows DNA-damaged hepatocytes to escape p53-induced apoptosis, the potent genome surveillance checkpoint, via *Bcl-G* silencing, which results in the fixation of mutations and promotion of future HCC development. In summary, G9a determines whether hepatocytes undergo apoptosis or survive during DNA damage response via the regulation of p53 transactivation.

Accumulating evidence suggests that epigenetic dysregulation plays an important role in human carcinogenesis.^5^ Histone modifiers play crucial roles in regulating oncogenes and tumor suppressor genes in various cancer types, including HCC. The establishment of HCC is a complex process which involves the accumulation of multiple gene mutations. However, it is unclear how mutated hepatocytes escape p53-dependent genome surveillance and progress to HCC. In this study, we reveal the role of histone methyltransferase G9a in hepatocarcinogenesis in vivo. In a pro-carcinogen DEN-induced murine HCC model, G9a prevented DNA-damaged hepatocytes from undergoing apoptosis by modulating p53 transactivation (Fig. 6). This is a novel finding of the role of epigenetic regulation to liver cancer initiation.

We showed, by analyzing a TCGA data set, that several histone methyltransferases, including *SETDB1* and *G9a*, are overexpressed in HCC. Interestingly, the loci of both genes, chromosome 6p21 of *G9a* and chromosome 1q21 of *SETDB1*, are amplified in human HCC^22,33^, suggesting that upregulation of *G9a* may be due to copy number amplification, as in the case of *SETDB1*. H3K9 methyltransferases including G9a, GLp, SETDB1, and Suv39h1, functionally cooperate as components of the transcriptional megacomplex^34^. Since they are important in liver carcinogenesis^8,9,16^, we suggest that the coordinated activity of H3K9 methyltransferase may play a role in liver tumor initiation. Indeed, we observed a residual increase in H3K9 levels regardless of G9a inhibition in DNA-damaged human hepatocytes, suggesting that other H3K9 methyltransferases may be involved.

Surviving DNA damage, such as that induced by reactive oxygen species or radiation exposure, involves the cells; ability to adapt to DNA damage. However, these cellular adaptations may enhance the viability of premalignant cells and their transformation to tumor cells. One group reported that G9a is required for DNA damage repair and allowed cancer cells to survive under DNA damage^21^. Another group demonstrated that G9a is recruited to DNA damage sites with other repressive chromatin proteins such as enhancer of zeste homolog 2 (EZH2) and DNA methyltransferase (DNMT) and maintained transcriptional silencing of tumor suppressor genes in colorectal cancer^35^. These reports indicate the important roles for G9a in transcriptional repression and tumor initiation under DNA damage. Consistent with this notion, *G9a* ablation or pharmacological inhibition resulted in enhanced apoptosis in DNA-damaged hepatocytes and attenuated liver tumor initiation. Our data emphasize the pro-tumorigenic role of G9a by suppressing DNA damage-induced apoptosis in mammalian cells, including hepatocytes.

Transcriptional analysis revealed that G9a silenced Bcl-G expression, a pro-apoptotic member of the Bcl-2 family. In humans, there are two BCL-G isoforms, BCL-G_S_ and BCL-G_L_, generated by alternative splicing^36^. Both isoforms are related to the induction of apoptosis, with BCL-G_L_ being widely expressed in adult human tissues, whereas BCL-G_S_ is found only in the testicles. In mice, only one form of Bcl-G exists, which is homologous to human BCL-G_L_ and is expressed in similar tissues^37^. Studies on breast and colorectal cancers suggest that BCL-G has a tumor-suppressive function^38,39^. *Bcl-G* expression in HCC was less than half of that in the surrounding NT liver in our cohort (data not shown). Moreover, we found that overexpression of BCL-G in hepatoma cell lines in vitro induces apoptotic changes, suggesting its role as a tumor suppressor in hepatocarcinogenesis. Taken together, our study demonstrates that G9a allows cell survival and future malignant transformation by silencing the expression of tumor-suppressing Bcl-G in DNA-damaged hepatocytes.

Recent papers have reported the functional link of G9a with p53 activity. G9a specifically methylates p53 at lysine 373, resulting in its inactivation^40^. Loss of G9a delayed malignant transition in progenitors of a murine chemical mutagen-induced squamous tumor through p53 overactivation^15^. These reports suggest that G9a promotes tumorigenesis by impairing p53 transactivation. Moreover, G9a and HDAC1 epigenetically repress p53 target genes by maintaining H3K9 methylation and histone deacetylation of the p53 target genes promoter^41^. Consistently, our ChIP experiments revealed that G9a regulates p53 recruitment onto its target pro-apoptotic gene, *Bcl-G*, in an H3K9-methylase dependent manner. In contrast, the regulation was not in the case of p21 promoter. It remains unclear how the specificity of G9a binding to p53 target genes is determined. Since histone modifiers regulate gene expression through interaction with chromatin remodeling complexes^29,42^, a chromatin remodeling mechanism or chromatin conformational changes may be involved in the processes. Further studies are needed to elucidate this point.

Finally, G9a inhibitors could provide additional effects when combined with other agents such as DNMT inhibitors^19,43^ or immune checkpoint inhibitors^43,44^, which are expected to be effective against HCC^45^. Our results might point to a new therapeutic strategy of combinatory therapies, including G9a inhibitors, against HCC.

## Materials and methods

### Human clinical samples

Surgically resected HCC samples were obtained from patients who underwent hepatectomy at the University of Tokyo between November 2013 and October 2014. These procedures were approved by the Ethical Committee for Clinical Research of our institution and written informed consent was obtained from each patient. The clinical diagnosis of all samples as HCC was confirmed by the Department of Pathology at the University of Tokyo Hospital.

### The cancer genome atlas (TCGA) dataset

373 HCC samples with both mutation and mRNA data were obtained from TCGA via cBioportal (http://www.cbioportal.org/). Among them, putative copy-number alterations data calculated by GISTIC was available in 360 cases.

### Quantitative real-time PCR (qRT-PCR)

Total RNA was extracted from frozen clinical liver specimens, HCC cell lines, and mouse livers, using NucleoSpin RNAII (Takara, Shiga, Japan) or ISOGEN-II (Nippon Gene, Tokyo, Japan). The purified RNA was reverse transcribed using the ImProm_II Reverse Transcription System (Promega, Madison, WI, USA) and amplified by reverse transcription PCR. The qRT-PCR analysis was performed using a PCR mixture containing a complementary DNA sample, forward and reverse primers, and THUNDERBIRD SYBR qPCR Mix (Toyobo, Osaka, Japan), using the StepOnePlus Real-Time PCR System (Applied Biosystems, Foster City, CA, USA) according to the manufacturer’s instructions. Values were internally normalized against *β-actin* mRNA expression. The primer sequences are listed in Supplementary Table 1.

### Cell lines and treatment

The human cell lines Huh1, Huh6, Huh7, HLE, HLF, JHH1, JHH4, and JHH7 were obtained from the Japanese Collection of Research Bioresouces (JCRB; Osaka, Japan). HepG2 and PLC/PRF/5 were obtained from RIKEN Cell Bank (RCB; Tsukuba, Japan). Hep3B and SkHep1 were obtained from American Type Culture Collection (ATCC; Manassas, VA, USA). Human normal hepatocyte was obtained from Applied Cell Biology Research Institute (ACBRI; Kirkland, WA, USA). JHH1, JHH6, and JHH7 were maintained in William’s E Medium (Sigma, St Louis, MO, USA) containing 10% heat-inactivated fetal bovine serum (FBS). Other cell cultures were maintained in Dulbecco’s modified Eagle’s medium (DMEM; Sigma) containing 10% heat-inactivated FBS. All the cells were incubated at 37 °C and 5% CO_2_.

### Animal experiments

Floxed G9a (*G9a*^flox/flox^) mice were generated as described previously^10,46^ and *Alb-Cre* mice were purchased from The Jackson Laboratory^47^. Mice were maintained in a temperature- and light-controlled facility, and permitted ad libitum regular chow diet and autoclaved water. All mice were backcrossed with the C57BL/6 strain and the male progeny were analyzed. All the experiments were performed in accordance with protocols approved by the Animal Ethics Committee of the University of Tokyo.

HCC was induced as previously described^23^. 15-day-old WT and *G9a*^ΔHep^ mice were injected intraperitoneally (i.p.) with DEN (Sigma, St. Louis, MO, USA) (25 mg/kg) alone or in combination with 22 weekly injections of CCl_4_ (Wako, Osaka, Japan) (0.5 ml/kg i.p., dissolved in corn oil). To evaluate acute effects of G9a in damaged hepatocytes, 8-week-old WT and *G9a*^ΔHep^ mice were treated with DEN (100 mg/kg i.p.) and sacrificed 48 h after DEN administration. In vivo G9a inhibitor treatment was performed as previously described^48^. WT mice were injected i.p. with G9a inhibitor UNC0642 (5 mg/kg) for 10 days before and after DEN administration. Randomization or blinding of animal experiments were not possible.

### Primary hepatocyte isolation

Primary hepatocytes were isolated from mouse livers as previously described^49^. Briefly, 40 μg/ml Liberase (Roche, Grenzacherstrasse, Basel, Switzerland) was perfused through the liver. Digested liver was passed through a 40 μm cell strainer, and centrifuged several times at 50 *g* for 1 min to remove nonparenchymal cells.

### Immunoblotting

Whole-liver protein homogenates or cell lysates were prepared. Immunoblot was performed as previously described^29^. The primary antibodies used were against β-actin (Sigma, A5441), G9a (R&D Systems, Minneapolis, MN, USA, PP-A8320A-00), H3K9me2 (Abcam, Cambridge, UK, ab1220), Histone H3 (Abcam, ab1791), Bcl-G (Thermo Fisher Scientific, Waltham, MA, USA, PA5-20029), Cleaved-PARP (Cell Signaling, Danvers, MA, USA, 9541: human, 9544: mouse), Cleaved-Caspase 3 (Cell Signaling, 9661), Flag (Sigma, F1804), γH2AX (Abcam, ab2893), p53 (Cell Signaling, 2524), Phospho-p53 (Ser 15) (Cell signaling, 9284). Immunoblot signals were analyzed using Image J software and relative protein expression levels were internally normalized against β-actin expression levels.

### Histology and immunohistochemistry

Mouse livers were fixed with 4% paraformaldehyde and embedded in paraffin. Tissue sections were hematoxylin and eosin (H&E) stained for pathological evaluation. Immunohistochemistry was performed using Histofine Mousestain Kit (Nichirei Bioscience, Tokyo, Japan). The slides were subjected to heat mediated antigen retrieval using 10 mM citrate buffer (pH 6.0) and incubated overnight at 4 °C with the indicated primary antibodies. The antibodies used were against H3K9me2 (Abcam, ab1220), Ki67 (Abcam, ab15580), Cleaved-Caspase3 (Cell Signaling, 9661), G9a (R&D Systems, PP-A8320A-00), γH2AX (Abcam, ab2893), p53 (Leica Biosystems, Wetzlar, Germany, P53-CM5P-L), Bcl-G (Thermo Fisher Scientific, PA5-20029).

### Serum alanine aminotransferase (ALT) measurement

Serum samples for ALT measurement were collected after a 16 h starvation (SRL, Tokyo, Japan).

### Microarray analysis

GeneChip Mouse Genome 430 2.0 Arrays were performed according to the manufacturer’s protocol (Affymetrix, Santa Clara, CA, USA). Total RNA was extracted from non-tumorous (NT) liver of 30-week-old WT and G9a^ΔHep^ mice treated with DEN followed by 22 repeated CCl_4_ administration (each, *n* = 2). Probe design files and microarray data have been submitted to the National Center for Biotechnology Information Gene Expression Omnibus database under accession number GSE147061.

### Plasmids, short hairpin RNA (shRNA), and transfection

The human BCL-G expression plasmid, pCAGGS human flag-BCL-G, was kindly provided by Dr. Toyomasa Katagiri (Division of Genome Medicine, Institute of Advanced Medical Sciences, Tokushima University, Japan)^38^ The lentiviral-based knockdown plasmid expressing the specific shRNA of BCL-G was purchased from Open Biosystems (Huntsville, AL, USA). The cells were transfected using the Effectene Transfection Reagent (Qiagen, Hilden, Germany) according to the manufacturer’s instructions.

### Flow cytometry

For cell cycle distribution analysis, the hepatocyte lines were collected after 24 h of treatment with a G9a inhibitor UNC0638 or mock. The cells were fixed in 1 mL ethanol (70 %) at 4 °C for 30 min and were resuspended in phosphate buffered saline containing 50 μg/mL propidium iodide (PI) and incubated at room temperature for 30 min before analysis.

For apoptosis detection assay, the hepatocyte lines were collected 24 h after treatment with ultraviolet B (UVB) irradiation (300 J/m^2^) by a UVB lamp (UVP, Upland, CA, USA) or hydrogen peroxide (2 mM, lasting 24 h). The cells were stained with 5 μL of FITC Annexin V (BD Biosciences, Franklin Lakes, NJ, USA) and PI (50 μg/mL) for 15 min in the dark. PI negative and Annexin V positive cells were considered early apoptotic; PI and Annexin V positive cells were considered to be in late apoptosis.

Data analysis and acquisition were performed using the Guava® EasyCyte™ Plus Flow Cytometry System and the Guava Express Pro Software (Guava Technologies, Hayward, CA, USA).

### Chromatin immunoprecipitation (ChIP)

Chromatin immunoprecipitation were performed as previously described^29,50^. Fifty milligrams of mouse livers were crosslinked in 1% formaldehyde/Phosphate-buffered saline for 15 min at room temperature, which was quenched by 0.125 M glycine for 5 min. The tissue was homogenized, resuspended in cell lysis buffer (10 mM Hepes pH 7.9, 0.5% NP-40, 1.5 mM MgCl_2_, 10 mM KCl, 0.5 mM DTT), and incubated at 4 °C for 4 h. The nuclei were resuspended in nuclear lysis buffer (20 mM Hepes pH7.9, 25% Glycerol, 0.5% NP-40, 0.42 M NaCl, 1.5 mM MgCl_2_, 0.2 mM ethylenediaminetetraacetic acid), and sonicated for 15 cycles (30 s ON/30 s OFF) using Bioruptor UCD-250 (Cosmo Bio, Carlsbad, CA, USA) to yield DNA fragments approximately of 500 bp. The soluble chromatin lysate was then immunoprecipitated using antibodies against G9a (Cell Signaling, 3306), p53 (Cell Signaling, 2524), and H3K9me2 (Abcam, ab1220) or control IgG (rabbit: Cell Signaling, 2729, mouse: Santa Cruz, sc-2025).

The ChIPed DNA was purified using QIA quick PCR purification kit (Qiagen). Results from each immunoprecipitation were presented as input percentage. The primer sequences for the target regions are available in Supplementary Table 2.

### Statistical analyses

All statistical analyses were performed using R software (version 3.6.1; R Development Core Team, Vienna, Austria). All the results are expressed as mean ± standard error of the mean (SEM) of at least three independent experiments. Statistical significance was determined by the two-tailed Student’s *t* test and Fisher’s exact probability test. *p* values < 0.05 were considered as statistically significant.

## Supplementary information

Supplementary Tables

Supplementary Figure Legends

Figure S1

Figure S2

Figure S3

## Supplementary information

**Supplementary Information** The online version contains supplementary material available at (10.1038/s41419-020-03381-1).

## References

[1] Villanueva A (2019). Hepatocellular carcinoma. N. Engl. J. Med.

[2] Mann DA (2014). Epigenetics in liver disease. Hepatology.

[3] Totoki Y (2014). Trans-ancestry mutational landscape of hepatocellular carcinoma genomes. Nat. Genet.

[4] Fujimoto A (2016). Whole-genome mutational landscape and characterization of noncoding and structural mutations in liver cancer. Nat. Genet.

[5] Dawson MA, Kouzarides T (2012). Cancer epigenetics: from mechanism to therapy. Cell.

[6] Cedar H, Bergman Y (2009). Linking DNA methylation and histone modification: patterns and paradigms. Nat. Rev. Genet.

[7] Au SL (2012). Enhancer of zeste homolog 2 epigenetically silences multiple tumor suppressor microRNAs to promote liver cancer metastasis. Hepatology.

[8] Fan DN (2013). Histone lysine methyltransferase, suppressor of variegation 3-9 homolog 1, promotes hepatocellular carcinoma progression and is negatively regulated by microRNA-125b. Hepatology.

[9] Fei Q (2015). Histone methyltransferase SETDB1 regulates liver cancer cell growth through methylation of p53. Nat. Commun..

[10] Tachibana M (2002). G9a histone methyltransferase plays a dominant role in euchromatic histone H3 lysine 9 methylation and is essential for early embryogenesis. Genes Dev..

[11] Shinkai Y, Tachibana M (2011). H3K9 methyltransferase G9a and the related molecule GLP. Genes Dev..

[12] Ding J (2013). The histone H3 methyltransferase G9A epigenetically activates the serine-glycine synthesis pathway to sustain cancer cell survival and proliferation. Cell Metab..

[13] Cha ST (2016). G9a/RelB regulates self-renewal and function of colon-cancer-initiating cells by silencing Let-7b and activating the K-RAS/beta-catenin pathway. Nat. Cell Biol..

[14] Tu WB (2018). MYC Interacts with the G9a Histone Methyltransferase to Drive Transcriptional Repression and Tumorigenesis. Cancer Cell.

[15] Avgustinova A (2018). Loss of G9a preserves mutation patterns but increases chromatin accessibility, genomic instability and aggressiveness in skin tumours. Nat. Cell Biol..

[16] Wei L (2017). Histone methyltransferase G9a promotes liver cancer development by epigenetic silencing of tumor suppressor gene RARRES3. J. Hepatol..

[17] Hu Y (2019). G9a and histone deacetylases are crucial for Snail2-mediated E-cadherin repression and metastasis in hepatocellular carcinoma. Cancer Sci..

[18] Yokoyama M (2017). Histone lysine methyltransferase G9a is a novel epigenetic target for the treatment of hepatocellular carcinoma. Oncotarget.

[19] Barcena-Varela M (2019). Dual Targeting of Histone Methyltransferase G9a and DNA-Methyltransferase 1 for the Treatment of Experimental Hepatocellular Carcinoma. Hepatology.

[20] Wang Z, Li Z, Ye Y, Xie L, Li W (2016). Oxidative Stress and Liver Cancer: Etiology and Therapeutic Targets. Oxid. Med Cell Longev..

[21] Yang Q (2017). G9a coordinates with the RPA complex to promote DNA damage repair and cell survival. Proc. Natl Acad. Sci. USA.

[22] Wong CM (2016). Up-regulation of histone methyltransferase SETDB1 by multiple mechanisms in hepatocellular carcinoma promotes cancer metastasis. Hepatology.

[23] Dapito DH (2012). Promotion of hepatocellular carcinoma by the intestinal microbiota and TLR4. Cancer Cell.

[24] Kang JS, Wanibuchi H, Morimura K, Gonzalez FJ, Fukushima S (2007). Role of CYP2E1 in diethylnitrosamine-induced hepatocarcinogenesis in vivo. Cancer Res..

[25] Maeda S, Kamata H, Luo JL, Leffert H, Karin M (2005). IKKbeta couples hepatocyte death to cytokine-driven compensatory proliferation that promotes chemical hepatocarcinogenesis. Cell.

[26] Bergmann J (2017). IL-6 trans-signaling is essential for the development of hepatocellular carcinoma in mice. Hepatology.

[27] Siliciano JD (1997). DNA damage induces phosphorylation of the amino terminus of p53. Genes Dev..

[28] Miled C, Pontoglio M, Garbay S, Yaniv M, Weitzman JB (2005). A genomic map of p53 binding sites identifies novel p53 targets involved in an apoptotic network. Cancer Res.

[29] Nakatsuka T (2017). Impact of histone demethylase KDM3A-dependent AP-1 transactivity on hepatotumorigenesis induced by PI3K activation. Oncogene.

[30] Tateishi K, Okada Y, Kallin EM, Zhang Y (2009). Role of Jhdm2a in regulating metabolic gene expression and obesity resistance. Nature.

[31] Yamamoto S (2013). Histone demethylase KDM4C regulates sphere formation by mediating the cross talk between Wnt and Notch pathways in colonic cancer cells. Carcinogenesis.

[32] Nakagawa H (2011). Apoptosis signal-regulating kinase 1 inhibits hepatocarcinogenesis by controlling the tumor-suppressing function of stress-activated mitogen-activated protein kinase. Hepatology.

[33] Moinzadeh P, Breuhahn K, Stutzer H, Schirmacher P (2005). Chromosome alterations in human hepatocellular carcinomas correlate with aetiology and histological grade-results of an explorative CGH meta-analysis. Br. J. Cancer.

[34] Fritsch L (2010). A subset of the histone H3 lysine 9 methyltransferases Suv39h1, G9a, GLP, and SETDB1 participate in a multimeric complex. Mol. Cell.

[35] Xia L (2017). CHD4 has oncogenic functions in initiating and maintaining epigenetic suppression of multiple tumor suppressor genes. Cancer Cell.

[36] Guo B, Godzik A, Reed JC (2001). Bcl-G, a novel pro-apoptotic member of the Bcl-2 family. J. Biol. Chem..

[37] Giam M (2012). Detection of Bcl-2 family member Bcl-G in mouse tissues using new monoclonal antibodies. Cell Death Dis..

[38] Lin ML, Park JH, Nishidate T, Nakamura Y, Katagiri T (2007). Involvement of maternal embryonic leucine zipper kinase (MELK) in mammary carcinogenesis through interaction with Bcl-G, a pro-apoptotic member of the Bcl-2 family. Breast Cancer Res.

[39] Nguyen PM (2019). Loss of Bcl-G, a Bcl-2 family member, augments the development of inflammation-associated colorectal cancer. Cell Death Differ.

[40] Huang J (2010). G9a and Glp methylate lysine 373 in the tumor suppressor p53. J. Biol. Chem..

[41] Heo K (2013). Cell-penetrating H4 tail peptides potentiate p53-mediated transactivation via inhibition of G9a and HDAC1. Oncogene.

[42] Abe Y (2015). JMJD1A is a signal-sensing scaffold that regulates acute chromatin dynamics via SWI/SNF association for thermogenesis. Nat. Commun..

[43] Segovia C (2019). Inhibition of a G9a/DNMT network triggers immune-mediated bladder cancer regression. Nat. Med.

[44] Urrutia G (2020). Combined targeting of G9a and checkpoint kinase 1 synergistically inhibits pancreatic cancer cell growth by replication fork collapse. Mol. Cancer Res..

[45] Cheng AL, Hsu C, Chan SL, Choo SP, Kudo M (2020). Challenges of combination therapy with immune checkpoint inhibitors for hepatocellular carcinoma. J. Hepatol..

[46] Tachibana M, Nozaki M, Takeda N, Shinkai Y (2007). Functional dynamics of H3K9 methylation during meiotic prophase progression. EMBO J..

[47] Postic C, Magnuson MA (2000). DNA excision in liver by an albumin-Cre transgene occurs progressively with age. Genesis.

[48] Kim Y (2017). Targeting the histone methyltransferase G9a activates imprinted genes and improves survival of a mouse model of Prader-Willi syndrome. Nat. Med..

[49] Nakagawa H (2014). ER stress cooperates with hypernutrition to trigger TNF-dependent spontaneous HCC development. Cancer Cell.

[50] Yamamoto K (2016). Stromal remodeling by the BET bromodomain inhibitor JQ1 suppresses the progression of human pancreatic cancer. Oncotarget.

