# Supplementary material for: Inhibition of histone methyltransferase G9a attenuates liver cancer initiation by sensitizing DNA-damaged hepatocytes to p53-induced apoptosis
Source: Cell Death Dis. 2021 Jan 19;12(1):99. doi: 10.1038/s41419-020-03381-1 (PMC7815717; doi:10.1038/s41419-020-03381-1)
Supplement: Supplementary file 1 — Supplementary Tables [file 41419_2020_3381_MOESM1_ESM.docx]

**Supplementary Table 1.** **qRT-PCR primers**

| **Gene** | **Forward primer** | **Reverse primer** |
| --- | --- | --- |
| **Human** |  |  |
| *ACTB* | CTGGAACGGTGAAGGTGACA | AAGGGACTTCCTGTAACAACGCA |
| *G9a* | TCCGCTGATTTTCGAGTGTAAC | CCCGGTTCTTGCAGTTTCTC |
| *BCL-G* | AGCACGGTGGATGGGAAAAA | TGCTCCATCTGCCTTGTACG |
| **Mouse** |  |  |
| *Actb* | ATGTGGATCAGCAAGCAGGA | AAGGGTGTAAAACGCAGCTCA |
| *Cyp2e1* | ACTTTGGCCGACCTGTTCTT | TCATGCACTACAGCGTCCAT |
| *G9a* | AGGATGGCGAGGTTTACTGC | TGAAAACCCGGACAGGGATG |
| *Bcl-G* | CACGTGTCTGTCGGTCTCAT | AGTAATCAGGGCATCCCAGG |
| *p21* | TCCAGACATTCAGAGCCACAG | AAAGTTCCACCGTTCTCGGG |

**Supplementary Table 2. ChIP-qPCR primers**

| **Gene** | **Forward primer** | **Reverse primer** | **Target site** |
| --- | --- | --- | --- |
| *Bcl-G* | GAAAGTAGCGTGGCTTTGCTC | CTAGGCCAGTGGTAGGTGCT | 10kbp upstream of TSS |
|  | GGTCAAGACACTCAGGCCAA | CTTTGGAAGGCTCCTCTCGG | TSS |
|  | CCTCACTGGGTTGACACTCG | CATCGTGGAAGAGTCCGGTG | p53RE |
|  | CTGACCAAAATCCCGCACAC | GGGATGGGGACTGGTCTTTT | 10kbp downstream of TSS |
| *p21* | AGTGATACGGAGCCTGGAGA | GGCAAAAGCCAGCATTCCTT | 10kbp upstream of TSS |
|  | TCTCCCTTGGTCCCTTGGAT | ATTTTGCTGCTGGTCTCCGA | p53RE |
|  | TTTGTGGTGCTCTGGGAAGC | CTGAGTGCTGTGACCTCCTG | TSS |
|  | GTCTCACGAGAAGGGACAGC | ATTGAGCACCAGCTTTGGGG | 10kbp downstream of TSS |

Primer pairs were designed to recognize about 500bp sequence around p53 response element (RE), transcription start site (TSS), and the region 10kbp upstream or downstream of TSS in respective gene.
