# Supplementary material for: Inhibition of histone methyltransferase G9a attenuates liver cancer initiation by sensitizing DNA-damaged hepatocytes to p53-induced apoptosis
Source: Cell Death Dis. 2021 Jan 19;12(1):99. doi: 10.1038/s41419-020-03381-1 (PMC7815717; doi:10.1038/s41419-020-03381-1)
Supplement: Supplementary file 2 — Supplementary Figure Legends [file 41419_2020_3381_MOESM2_ESM.docx]

**Fig. S1 KMT and KDM expression levels in the TCGA data set.**

Relative expression levels of histone-lysine methyltransferase (KMT) and demethylase (KDM) in hepatocellular carcinoma using The Cancer Genome Atlas (TCGA) data set. *G9a* (*EHMT2*) expression is the second highest.

**Fig. S2 Pathological features of the formed tumor cells and the attenuation of DEN-induced hepatocarcinogenesis in *G9a*^ΔHep^ livers.**

(A) Representative pathological images of the liver tumors. The numbers of Ki67 and cleaved-Caspase 3 positive hepatocytes are comparable between WT and *G9a*^ΔHep^ liver tumors. T represents the liver tumor, and NT represents the surrounding normal liver tissue. Scale bars, 50µm; NS not significant, Student's *t*-test. (B) WT and *G9a*^ΔHep^ mice are subjected to diethylnitrosamine (DEN) (25 mg/kg i.p.) at day 15 postpartum and euthanized 38 months after DEN administration. The development of liver tumors is significantly attenuated in *G9a*^ΔHep^ mice (WT, n = 8; *G9a*^+/-^, n = 10; *G9a*^ΔHep^, n = 10; ** *P* < 0.01, Student’s *t*-test).

**Fig. S3 The effect of a G9a inhibitor UNC0638 in human normal hepatocyte cell lines.**

(A) UNC0638 (5µM) significantly attenuates the global levels of H3K9me2 in the hepatocyte lines. (B) Treatment with UNC0638 (5µM) for 24 h has no effect on the cell cycle status.
